# An App to Support Difficult Interactions Among Providers, Patients, and Families

**DOI:** 10.6004/jadpro.2015.6.5.8

**Published:** 2015-09-01

**Authors:** Joy V. Goldsmith, Elaine Wittenberg, Betty Ferrell

**Affiliations:** 1University of Memphis, Memphis, Tennessee; 2City of Hope Comprehensive Cancer Center, Nursing Research and Education, Duarte, California

Oncology advanced practitioners are responsible for effective communication regarding diagnosis, prognosis, and treatment options. The Institute of Medicine’s Report on Delivering High-Quality Cancer Care recommends that patients and their families be provided understandable information and decision aids to personalize information at key decision points along the continuum of cancer care (Institute of Medicine, 2013).

One of the most challenging aspects of clinical care is sensitive and timely communication with patients and families. With the explosion of applications (apps) for oncology providers (Doyle-Lindrud, 2014), there is an opportunity for increased technology use to facilitate patient/family caregiver communication.

The range of mobile health, or ’’mHealth,’’ apps is impressive, spanning care delivery, monitoring, diagnostics, training, and more (Kumar, Nilsen, Pavel, & Srivastava, 2013). The Healthcare Information Management Systems Society’s 2014 study on mobile device analytics finds that device usage has proliferated and that clinicians tend to view mHealth tools positively. Clinicians indicate that future technologies will have a positive impact on their communication with other clinicians and patients (Healthcare Information Management Systems Society, 2014).

Health-care technologies and patient care have evolved rapidly, whereas the communication utility and efficacy of mHealth technologies have lagged behind (Nagler et al., 2014). Too little is known about how health-care providers utilize these systems (Barakat, Woolrych, Sixsmith, Kearns, & Kort, 2013) and about how patient/provider communication can be supported with mHealth technologies. Understanding the communication value of these technologies must move beyond a nascent area of research (Sherry & Ratzan, 2012). Communication skills training for providers improves the quality of life of patients and improves patient satisfaction with the quality of care (Fukui, Ogawa, & Yamagishi, 2011; Visser & Wysmans, 2010).

The COMFORT communication curriculum (see Table 1) is the first theoretically grounded curriculum developed for teaching patient-centered communication (Wittenberg-Lyles, Goldsmith, Ferrell, & Ragan, 2012). The app described here was created approximately 18 months ago and is an mHealth translation of the COMFORT curriculum (www.pccinstitute.com).

**Table 1:**
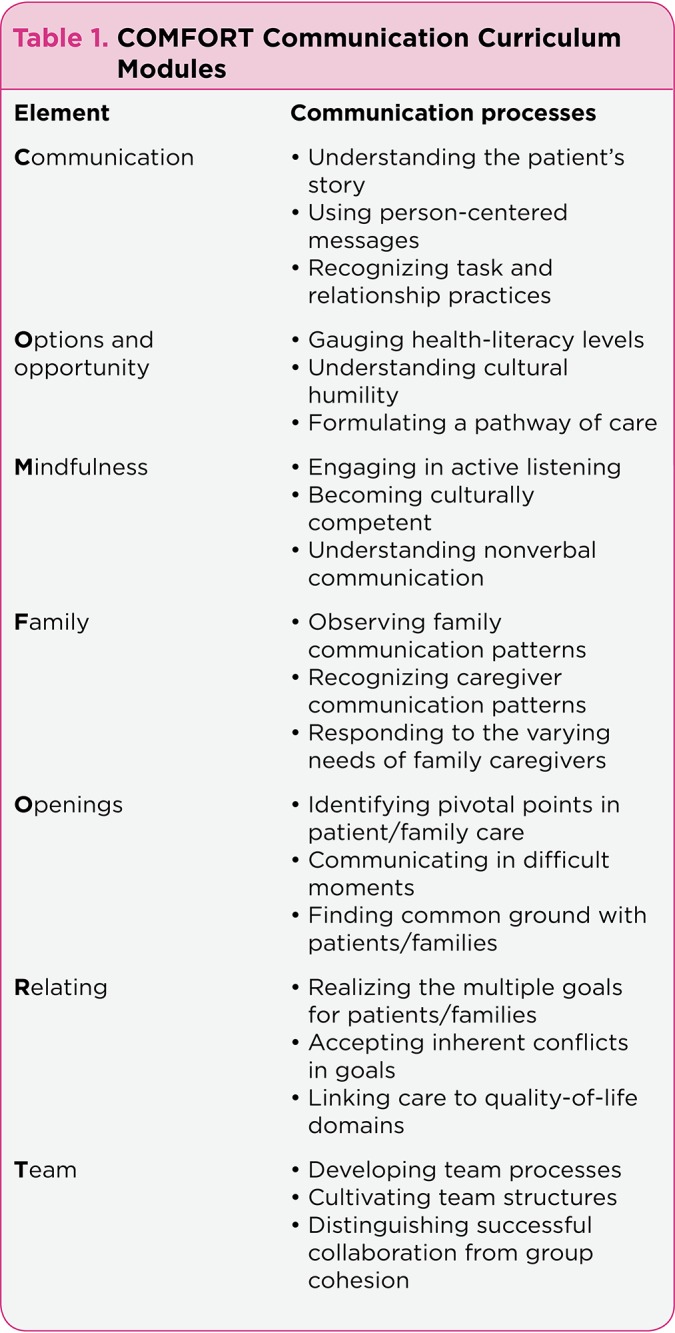
COMFORT Communication Curriculum Modules

To date, the Health Communication app is the only existing mHealth iOS (iPhone operating system) app that provides supportive communication tools for health-care providers encountering communication challenges with patients, families, and other team members. The content and design offer practice suggestions with language at a sixth-grade level to support advanced practitioners with communication resources and a simplistic design.

## Project Design and Method

We pursued a convenience sample of eight providers, including two physicians, two nurses, two social workers, and two chaplains. Inclusion criteria included professional practice at a cancer center and involvement in palliative care. Familiarity with handheld iOS devices was required. Two study team researchers administered testing with each participant in his or her preferred setting. This educational research activity was exempt under the institutional review board at the supporting university.

Participants were asked to complete several video-recorded tasks, which were assessed for completion attempts, length of time, and success/failure. Tasks included opening features of the app and browsing for specific information. Following task assessment, participants completed the Systems Usability Scale (SUS; Brooke, 1996) and a qualitative survey to assess the effectiveness, efficiency, and satisfaction of the app.

**Usability of the Health Communication iOS App**

Of the eight participants, six practice at a comprehensive cancer center in the western United States, one practices at a comprehensive pediatric cancer center in the mid-south, and one practices at a pediatric care center in the mid-south. Six of the eight providers reported using mHealth apps regularly.

Participants performed all tasks in 11 seconds or less. All tasks were accomplished successfully, with only one task requiring more than two attempts (mean); the remaining tasks were accomplished in 1.50 attempts or less (see Table 2).

**Table 2:**
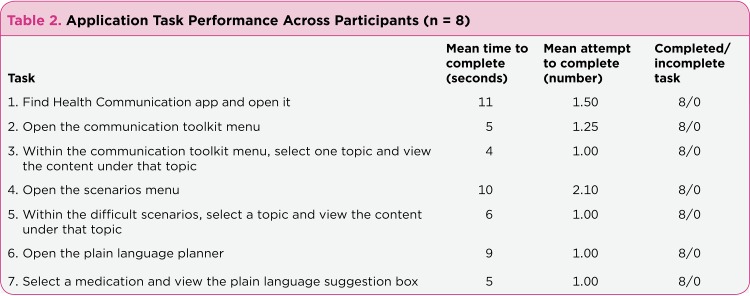
Application Task Performance Across Participants (n = 8)

The SUS measured the Health Communication app’s usability at a 91.56 on a scale of 0 to 100, achieving a grade of A (Bangor, Kortum, & Miller, 2009; see Table 3). Scaled converted responses that total an 80.3 or higher were graded as an A (the top 10% of scores) with the SUS tool. This is also the point at which users are more likely to recommend the product to a friend (Bangor et al., 2009).

**Table 3:**
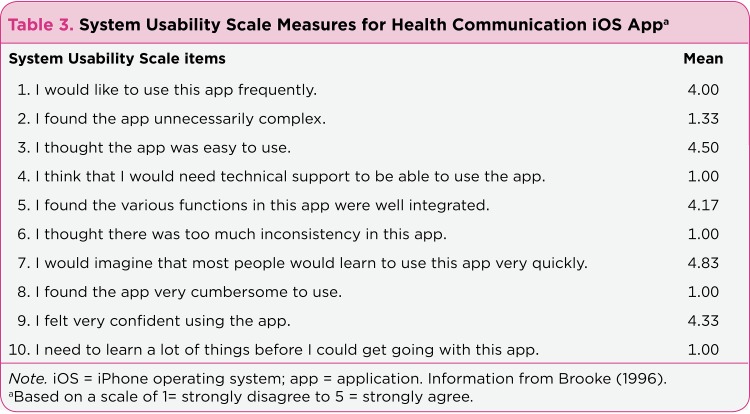
System Usability Scale Measures for Health Communication iOS App^a^

Qualitative responses revealed that all providers found the app easy to use and navigate and aesthetically satisfying. Similarly, all providers indicated they would use the app with patients and families as well as other team members (see Table 4). Physicians and nurses described the app as designed to "teach," "inform," and "educate," whereas social workers indicated the app could facilitate communication between physicians and patients. Chaplains described the app as "equipping" and "empowering" their clinical practice communication.

**Table 4:**
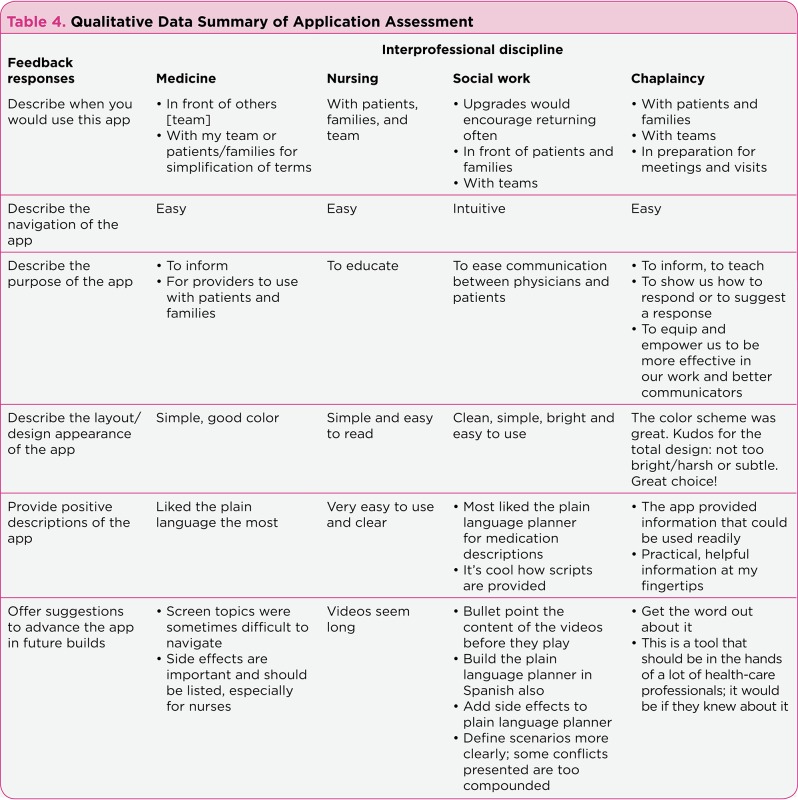
Qualitative Data Summary of Application Assessment

Positive descriptors of the app focused on the benefits of the plain language planner as well as the utility of having potential responses at hand for difficult encounters. Suggested changes for future builds included simplifying conflict topics, altering video clip content, and fortifying the plain language planner with content that includes side effects and language translations.

The Health Communication iOS app performed strongly on the SUS; task performance was 100% successful, with rapid completion in few attempts. Qualitative feedback showed collective agreement about its ease of use, layout, and navigation. Participant descriptions of the app confirmed its utility in providing useful content to the four provider areas (medicine, nursing, social work, chaplaincy) considered in this study.

## Tool Limitations and Future Directions

The app is currently available for iOS-platform devices only. To date, this mobile resource has been tested only with oncology health-care providers experienced in palliative care. Future intervention research with the app should examine an array of care specialties and contexts. In addition, future updates should incorporate unfolding research associated with the app’s toolkit, difficult scenarios, and plain language planner as they are studied and tested in clinical contexts.

## Conclusions

The Health Communication iOS app is a free intervention available from the iTunes store. The interactive possibilities of mHealth present limitless opportunities and challenges as we create, test, and restructure resources to deliver quality cancer care for patients and their families.
